# Comparative Effectiveness of Ustekinumab and Vedolizumab as Maintenance Therapy After Tacrolimus-Induced Improvement in Patients with Acute Severe Ulcerative Colitis: A Retrospective Cohort Study

**DOI:** 10.3390/jcm14155588

**Published:** 2025-08-07

**Authors:** Koji Kaku, Toshiyuki Sato, Jiro Takeuchi, Keiko Yokoyama, Soichi Yagi, Yasuhiro Takagi, Maiko Ikenouchi, Mikio Kawai, Koji Kamikozuru, Yoko Yokoyama, Tetsuya Takagawa, Toshihiko Tomita, Hirokazu Fukui, Shinichiro Shinzaki

**Affiliations:** 1 Department of Gastroenterology, School of Medicine, Hyogo Medical University, 1-1 Mukogawa, Nishinomiya 663-8501, Hyogo, Japan; ko-kaku@hyo-med.ac.jp (K.K.); tshnngn@hyo-med.ac.jp (T.S.); abc458017@gmail.com (K.Y.); so-yagi@hyo-med.ac.jp (S.Y.); suhir1017@gmail.com (Y.T.); ma-ikenouchi@hyo-med.ac.jp (M.I.); mkawai@hyo-med.ac.jp (M.K.); turuturu@hyo-med.ac.jp (K.K.); yoko0502@hyo-med.ac.jp (Y.Y.); tomita@hyo-med.ac.jp (T.T.); hfukui@hyo-med.ac.jp (H.F.); 2 Institute for Clinical and Translational Science, Nara Medical University, Nara 634-8522, Japan; jiroutakeuchi@gmail.com; 3 Department of Clinical Epidemiology, Hyogo Medical University, Nishinomiya 663-8501, Hyogo, Japan; 4 Center for Clinical Research and Education, Hyogo Medical University, Nishinomiya 663-8501, Hyogo, Japan; takagawa@hyo-med.ac.jp

**Keywords:** ulcerative colitis, acute severe ulcerative colitis, ustekinumab, vedolizumab, tacrolimus

## Abstract

**Background/Objectives:** Acute severe ulcerative colitis (ASUC) is often managed by tacrolimus induction therapy followed by maintenance therapy. We compared the effectiveness of ustekinumab versus vedolizumab as maintenance therapies after tacrolimus induced improvement in patients with ASUC. **Methods:** This single-center retrospective cohort study included patients with ASUC who received tacrolimus induction therapy followed by ustekinumab or vedolizumab between January 2018 and November 2024. The primary outcome was clinical remission at week 16. Secondary and exploratory outcomes included clinical remission at week 8, biologic persistence, and relapse risk. An inverse probability of treatment weighting (IPTW) analysis was performed using the following covariates: male sex, prior biologics or JAK inhibitors, partial Mayo score, CRP, and albumin. **Results:** Among 235 tacrolimus-treated patients, 29 received ustekinumab and 22 received vedolizumab. After IPTW adjustment, the clinical remission rates were significantly higher in the ustekinumab group at both week 8 (82.1% vs. 51.8%, *p* = 0.02) and week 16 (85.4% vs. 36.8%, *p* = 0.02). Biologic persistence was significantly higher in the ustekinumab group (*p* = 0.004), and ustekinumab significantly reduced the hazard of relapse in multivariable analyses (HR 0.42 [95% CI: 0.20–0.88], *p* = 0.02). **Conclusions:** Ustekinumab showed greater effectiveness than vedolizumab in terms of achieving remission at 16 weeks after tacrolimus induction therapy in patients with ASUC.

## 1. Introduction

Ulcerative colitis (UC) is a chronic inflammatory bowel disease that causes inflammation and ulcers in the colon and rectum [1]. Acute severe UC (ASUC) is a clinically serious condition that requires hospitalization and immediate medical therapy. Medical treatment failure frequently leads to colectomy [2].

Calcineurin inhibitor (CNI), including tacrolimus (TAC), rescue remains part of the therapeutic armamentarium for steroid-refractory acute severe ulcerative colitis (ASUC), although their adoption varies geographically. In Japan, oral TAC is explicitly recommended for severe UC failing intravenous corticosteroids [3]. In contrast, Western guidelines frame rescue therapy primarily as infliximab or cyclosporine [4,5,6] (Supplementary Table S1). TAC has demonstrated short-term efficacy in inducing clinical response or remission in ASUC patients [7]. Although some reports have shown that the long-term use of TAC may be safe and effective [8], the European Crohn’s and Colitis Organisation (ECCO) guidelines recommend discontinuing CNIs within six months and transitioning to thiopurines for remission maintenance [9], while the American Gastroenterological Association (AGA) also supports switching to thiopurines or vedolizumab (VED) as potential maintenance options [6]. Thiopurine-based maintenance therapy following TAC induction has been commonly used, but the effectiveness remains limited. The rate of total colectomy remains high, with over 33% of patients undergoing colectomy per year [10]. These challenges highlight the need to explore alternative maintenance therapies after CNI induction therapy in patients with ASUC.

Among potential therapeutic options, biologic agents such as VED and ustekinumab (UST) have shown promise. VED, a gut-selective antibody that targets α4β7 integrin, and UST, an antagonist of the p40 subunit of interleukin-12 and interleukin-23, are both effective in patients with UC [11,12] and being increasingly used after TAC induction. While both VED and UST have been studied as maintenance therapies after TAC induction, with several reports describing their individual outcomes [13,14,15], no head-to-head comparison between the two regimens has been reported yet. Therefore, we aimed to compare the effectiveness of VED and UST as maintenance therapies after TAC-induced clinical improvement in patients with ASUC, in order to clarify which agent may offer a more favorable treatment strategy in this setting.

## 2. Methods

### 2.1. Ethical Considerations

This study was approved by the Hyogo Medical University Institutional Ethics Review Board and was conducted in accordance with the Declaration of Helsinki. Because of the study’s retrospective nature, the Ethics Review Board waived the requirement for written informed consent and approved an opt-out approach. Opt-out protocol was used for the use or collection of participant data for research purposes. This consent procedure was reviewed and approved by the Hyogo Medical University Institutional Ethics Review Board (approval number: 4525; date of decision: 11 October 2023).

### 2.2. Study Design

We performed a single-center retrospective cohort study of patients with UC treated at Hyogo Medical University. The observation period was from the date of the first prescription of UST or VED to patients until March 2025.

### 2.3. Patients

We extracted the prescription codes for 1 mg and 0.5 mg TAC from the clinical medical records of patients with dates between January 2018 and November 2024, specifically targeting patients with UC who were treated with this medication. TAC was continued beyond the initial induction for two weeks; however, in some patients, it was maintained at a low trough concentration while monitoring disease activity, and treatment was gradually withdrawn when appropriate. If any renal function deterioration was observed, TAC was immediately discontinued. Renal function was closely monitored throughout TAC therapy, with serum creatinine and estimated glomerular filtration rate (eGFR) measured every visit. Patients were diagnosed with UC based on clinical assessments, endoscopy, and histology [3]. Patients with a diagnosis of ASUC at the time of hospital admission were selected for this study. We further refined the selection process by applying the inclusion and exclusion criteria described below. Eligible patients with ASUC at admission received TAC induction therapy before UST or VED maintenance therapy. Abdominal cross-sectional imaging and bedside flexible sigmoidoscopy were performed within the first 24 h to confirm disease extent and rule out complications. Pharmacologic deep-vein thrombosis (DVT) prophylaxis (e.g., low-molecular-weight heparin) was not routinely administered at our institution during the study period. Instead, each patient underwent standardized DVT risk assessment on admission, and those deemed at risk received mechanical prophylaxis—graduated compression stockings—and early mobilization. Furthermore, all patients underwent standard bacterial stool culture, and stool PCR (including *C. difficile* and enteropathogenic *Escherichia coli*) was performed when bacteria were detected on culture or antigen/toxin assays were positive. Although quantitative plasma or tissue CMV PCR and mucosal immunohistochemistry were performed in selected cases in which CMV reactivation is highly suspected by endoscopy and/or serum IgG/IgM testing, these assays were not systematically conducted in all patients because they were not covered by the national health insurance for the routine evaluation of ulcerative colitis flares in Japan during the study period.

### 2.4. Inclusion and Exclusion Criteria

Patients treated with TAC were included in this study. During this hospitalization period, patients were treated with rapid TAC induction therapy. Patients who received drugs other than UST or VED and those who received UST or VED after the discontinuation of TAC but were not followed-up for ≥16 weeks because of relocation were excluded.

### 2.5. Medications

TAC was orally administered at a dose of 6 mg/day in the fasting state with the goal of achieving a serum trough concentration of 10–15 ng/mL (high-dose therapy) within 1 week. The dose was subsequently adjusted every 2 days while monitoring blood levels. Following induction therapy with high-dose TAC for approximately 2 weeks, the dose of TAC was reduced to achieve a blood TAC concentration of 5–10 ng/mL. This approach aligns with the TAC induction method used in a previous report [16]. UST or VED was initiated as maintenance therapy starting from week 3 onward or when the TAC concentration dropped below 10 ng/mL; these patients transitioned to mild to moderate according to Truelove–Witts criteria [17,18]. For induction therapy, UST was intravenously administered at a dose determined by the patient’s body weight (≤55 kg: 260 mg; 55–85 kg: 390 mg; ≥85 kg: 520 mg). For maintenance therapy, UST was subcutaneously injected at a dose of 90 mg every 8 weeks. For induction therapy, VED was intravenously administered at a dose of 300 mg at weeks 0, 2, and 6. For maintenance therapy, VED was intravenously administered at a dose of 300 mg every 8 weeks.

### 2.6. Data Collection

The following characteristics were collected from the patients’ daily clinical practice records: age, sex, duration of disease, disease type, disease activity, laboratory data [C-reactive protein (CRP), white blood cell count (WBC), hemoglobin (Hb), platelet count (Plt), albumin (Alb)], concomitant thiopurine use, prior steroid use, prior use of a biologic and/or Janus kinase (JAK) inhibitor (e.g., infliximab, adalimumab, golimumab, tofacitinib, filgotinib, and upadacitinib), the initial dose of prednisolone at the time of administration of TAC, and the total dose of prednisolone after administration of TAC. The time in days from the start of TAC to the administration of the biologic was recorded. All adverse events were categorized using terms from the United States Food and Drug Administration [19,20]. Worsening of UC was not included as an adverse event.

### 2.7. Outcomes

The primary outcome was the clinical remission rate at week 16 following the administration of UST or VED in patients with TAC-induced improvement. The diagnosis of remission at week 16 was based on the results of a previous study of VED [14]. The evaluation periods were designated as week 16 to coincide with the third dose of UST and week 14 to coincide with the fourth dose of VED. The clinical remission rates at 16 weeks after initiating treatment with UST or VED were compared, where week 0 corresponded to the start of UST or VED treatment. The secondary outcome was the clinical remission rate at week 8. The evaluations planned for weeks 8 and 16 included a 2-week flexibility window preceding and following each dose. The evaluation at week 8 coincided with the second dose of UST and the third dose of VED. Exploratory outcomes included the biologic [UST and VED] persistence rate, surgical-free survival rate, and hospitalization-free survival rate at week 52. We also recorded the treatment used after the failure of UST and VED maintenance therapy.

### 2.8. Definitions

The definition of ASUC was based on Truelove–Witts criteria, although CRP was used instead of the erythrocyte sedimentation rate [17,18]. Clinical remission was defined as a partial Mayo score of less than two points and no more than one point for any individual subscore during the evaluation period [21]. TAC-induced improvement was defined as a reduction in disease activity from severe to mild–moderate according to the Truelove–Witts criteria [17,18].

Relapse was defined as an increase in the partial Mayo score by ≥3 points or by >1 point for any individual subscore, or a proposed switch to another drug. Steroid-refractory UC was defined as UC with relapse despite steroid treatment. UST and VED persistence rates were calculated by defining events as biologic discontinuation due to adverse events, relapse, or surgery. Cases where follow-up was not feasible, such as patient relocation, were handled as censored data in this analysis.

### 2.9. Statistical Analysis

We performed an inverse probability of treatment weighting (IPTW) analysis using the following covariates: male, prior use of biologics or JAK inhibitors, partial Mayo score, CRP, and albumin. All continuous variables are presented as the median and IQR, and were compared with the Mann–Whitney–Wilcoxon test. All categorical data were compared using Fisher’s exact test, and the chi-square test was used for IPTW-weighted analyses. Kaplan–Meier curves were generated for time-to-event data, defined as the time from the first dose of UST or VED until an event, such as the discontinuation of the biologic, hospitalization, or colectomy. In the Kaplan–Meier analysis, day 0 was designated as the date of starting biologic therapy, and time-to-event data were evaluated using the log-rank test. The Kaplan–Meier estimator was used to determine the event-free survival rates. To assess the risks of relapse throughout the observation period, we performed univariable and multivariable analyses using the Cox proportional hazards model. We also conducted multivariable analyses including the partial Mayo score at the time of UST or VED administration, the use of UST, and the interval between the initiation of TAC and biologic administration. Two-tailed *p* values of <0.05 were considered statistically significant. All statistical analyses were performed using R and EZR system version 1.61 (Saitama Medical Center, Jichi Medical University, Saitama, Japan) [22].

## 3. Results

### 3.1. Patient Demographics and Characteristics

**Figure 1:**
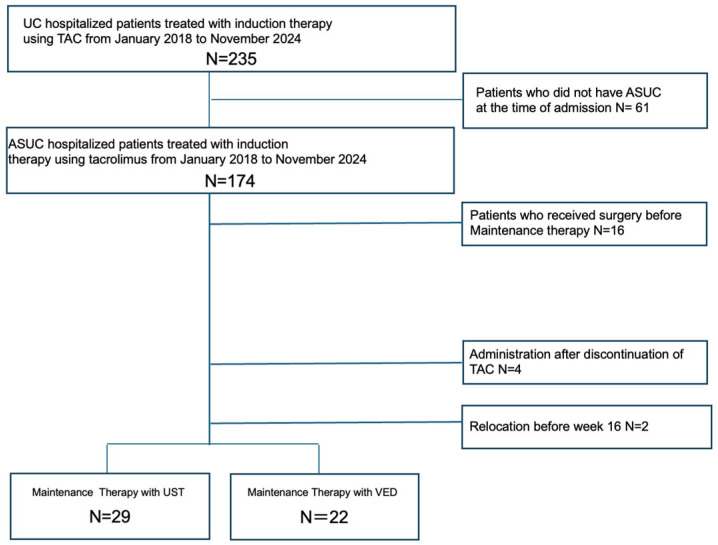
Patient flow chart. Abbreviations: ASUC, acute severe ulcerative colitis; TAC, tacrolimus; UC, ulcerative colitis; UST, ustekinumab; VED, vedolizumab.

A total of 235 hospitalized patients with UC underwent TAC induction therapy. We excluded 61 patients who did not meet the criteria for ASUC at admission, 16 patients received surgery before maintenance therapy, 101 patients did not receive UST or VED maintenance therapy, 4 patients started UST or VED after discontinuing TAC, and 2 patient relocated before week 16. Therefore, 51 patients were included in the final analysis, comprising 29 patients treated with UST and 22 patients treated with VED (Figure 1).

Table 1 shows that the baseline characteristics of admission before TAC induction therapy. The number of bloody stools at the time of admission in the UST group was higher than in the VED group [*p* = 0.01] [Table 1].

Table 2 and Table 3 summarize the baseline characteristics of patients after TAC-induced improvement and before the first UST/VED dose. Albumin levels at the time of administration tended to be lower in the UST group than in the VED group (*p* = 0.06). However, this difference was attenuated after the IPTW analysis (UST group: 3.4 [3.0, 4.0] g/dL; VED group: 3.9 [3.5, 4.1] g/dL; *p* = 0.10). After the IPTW analysis, the proportion of male patients remained significantly higher in the UST group (UST group: 18.8 [68.8%]; VED group: 8.1 [36.8%]; *p* = 0.04), and there was a trend toward a higher frequency of pancolitis in the UST group (UST group: 27.4 [100%]; VED group: 20.7 [94.1%]; *p* = 0.08).

The median follow-up was lower in the UST group than in the VED group [29.9 months (IQR 21.3–36.7) vs. 44.3 months (IQR 26.2–66.8) (*p* = 0.02)]. At the 6-month time point after the administration of TAC, the TAC concentrations were comparable between the VED and UST groups, with a median (IQR) concentration of 5.1 (3.3–6.0) in the VED group and 4.8 (3.1–5.9) in the UST group, showing no significant difference (*p* = 0.77). Similarly, serum creatinine levels and estimated glomerular filtration rate (eGFR) were not significantly different between groups [creatinine level: UST: 0.82 (0.74–0.90), VED: 0.79 (0.67–0.85), *p* = 0.27; eGFR: UST: 76.4 (70.0–89.2), VED: 74.5 (65.8–85.3), *p* = 0.77]. One patient in each group was concomitantly treated with 5 mg prednisolone; however, prednisolone was tapered off in 2 days for the patient in the UST group and in 3 days for the patient in the VED group. No patients were receiving systemic steroids at week 16. We also evaluated prior drug exposure counts only among patients with a history of use for each drug category. For anti-TNF agents, the median number of prior uses was 1 (IQR: 1–1) in the UST group and 1 (IQR: 1–2) in the VED group. For JAK inhibitors, the median number of prior uses was 1 (IQR: 1–1) in both UST and vedolizumab. For anti-integrin agents, the median number of prior uses was 1 (IQR: 1–1) in the UST group, while no patients in the VED group had previously received anti-integrin agents. For UST, the median number of prior uses was 1 (IQR: 1–1) in both UST and VED groups.

### 3.2. Primary and Secondary Outcomes

**Figure 2:**
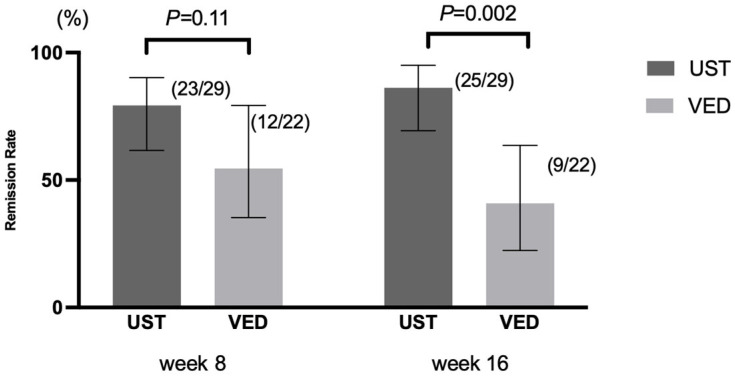
Clinical remission rates at weeks 8 and 16 before the IPTW analysis: UST showed a higher remission rate at week 16 (86.2% vs. 40.9%, *p* = 0.002), but no significant difference at week 8 (79.3% vs. 54.5%, *p* = 0.11). Error bars indicate 95% CI. Abbreviations: CI, confidence interval.

**Figure 3:**
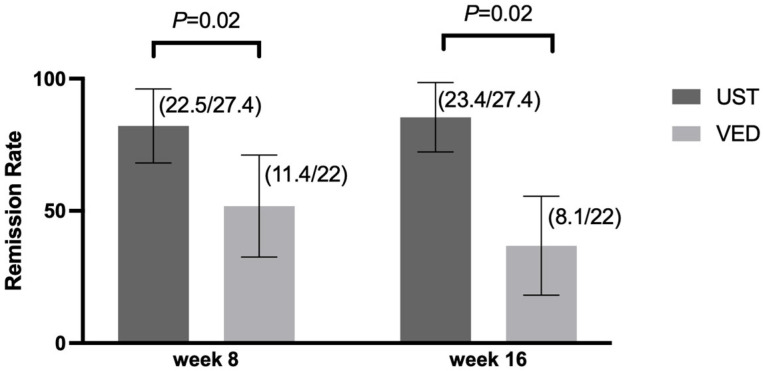
Clinical remission rates at weeks 8 and 16 after the IPTW analysis: UST showed significantly higher remission rates at both week 8 (82.1% vs. 51.8%, *p* = 0.02) and week 16 (85.4% vs. 36.8%, *p* = 0.02). Error bars indicate 95% CI.

At week 16, the clinical remission rate was significantly higher in the UST group (25 of 29 patients, 86.2% [95% confidence interval {CI}: 69.4–95.0%]) than in the VED group (9 of 22 patients, 40.9% [95% CI: 22.4–62.6%]) (*p* = 0.002) (Figure 2). However, the clinical remission at week 8 was not significantly different between the UST and VED groups (79.3% [95% CI: 61.6–90.2%] vs. 54.5% [95% CI: 33.7–73.7%], *p* = 0.11) (Figure 2). After the IPTW analysis, the clinical remission at weeks 8 and 16 was significantly higher in the UST than the VED group (82.1% [95% CI: 64.2–92.2%] vs. 51.8% [95% CI: 32.3–70.8%], *p* = 0.02 at week 8, and 85.4% [95% CI: 67.9–94.2%] vs. 36.8% [95% CI: 20.1–57.5%], *p* = 0.02 at week 16) (Figure 3).

### 3.3. Exploratory Outcomes

**Figure 4:**
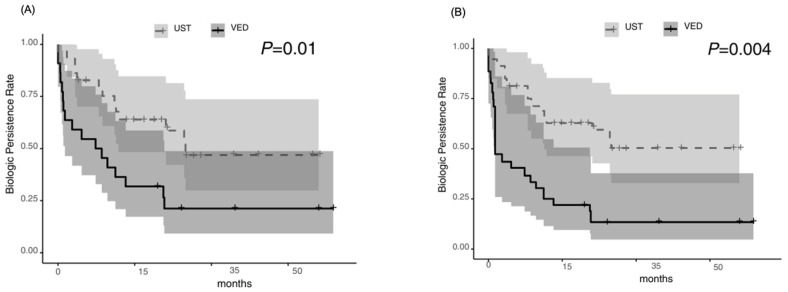
Biologic persistence: significantly higher in the UST group [*p* = 0.01 before the IPTW analysis (**A**), and *p* = 0.004 after the IPTW analysis (**B**)].

**Figure 5:**
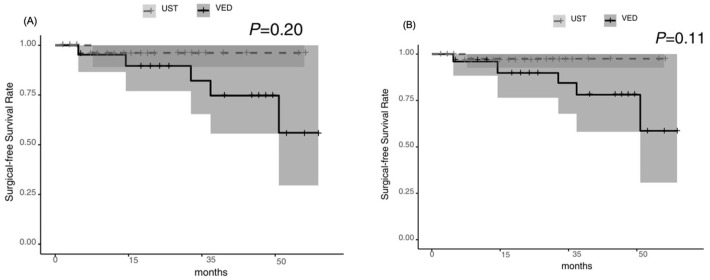
Surgical-free survival: no significant difference between the UST and VED groups [*p* = 0.20 before the IPTW analysis (**A**), and *p* = 0.11 after the IPTW analysis (**B**)].

**Figure 6:**
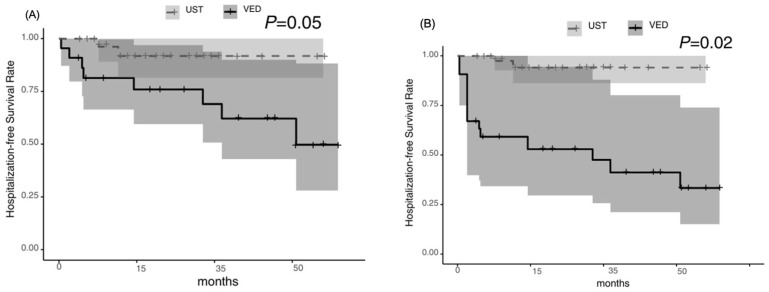
Hospitalization-free survival: tended to be higher in the UST group before the IPTW analysis (*p* = 0.05) (**A**) but was significantly higher in the UST group after the IPTW analysis (*p* = 0.02) (**B**).

The biologic persistence rate was significantly higher in the UST group than in the VED group [*p* = 0.01], with cumulative rates of 74.9% and 40.9%, respectively, at week 52. The median persistence time was 27.7 months [95% CI: 13.1–not applicable] for UST compared with 8.8 months [95% CI: 1.5–23.1] for VED (Figure 4A). The surgical-free survival rate was not significantly different between the two groups [*p* = 0.20]; however, the hospitalization-free survival rate of UST tended to be higher than that of VED [*p* = 0.05], with cumulative rates of 95.2% versus 95.8% and 95.8% versus 81.3%, respectively, at week 52 (Figure 5A and Figure 6A). After the IPTW analysis, the biologic persistence rate was still significantly higher in the UST group than in the VED group [*p* = 0.004] (Figure 4B), and hospitalization-free survival rate of UST was higher than that of the VED group [*p* = 0.02] (Figure 6B). The surgical-free survival rate was not significantly different between the two groups [*p* = 0.11] (Figure 5B).

### 3.4. Subsequent Treatments Following Relapse Within 1 Year of UST or VED Maintenance Therapy

**Figure 7:**
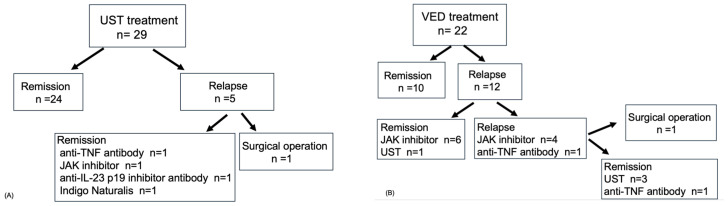
Subsequent treatments after failure of maintenance therapy. (**A**) Subsequent treatments after failure of maintenance therapy with UST within 1 year. (**B**) Subsequent treatments after failure of maintenance therapy with VED within 1 year. Abbreviations: JAK, Janus kinase; TNF, tumor necrosis factor; UST, ustekinumab; VED, vedolizumab.

Figure 7A shows the subsequent treatments following relapse in patients treated with UST. Of the 29 patients, 24 achieved remission, while 5 required additional therapies. Ultimately, one patient underwent surgical treatment by 1 year or by the end of the follow-up period. Figure 7B shows the flow of the subsequent treatments following relapse in patients treated with VED. Of the 22 patients, 10 achieved remission, while 12 required additional therapies. Ultimately, one patient underwent surgical treatment by 1 year or by the end of the follow-up period.

### 3.5. Risk Factors for Relapse Identified by Univariable and Multivariable Cox Proportional Hazards Models Before and After IPTW Adjustment

Table 4 provides the results of univariable analyses of factors associated with relapse. The use of UST significantly reduced the hazard of relapse in the univariable analyses (HR: 0.39 [95% CI: 0.19–0.81], *p* = 0.001). In multivariable analyses, the use of UST was significantly protective (HR: 0.42 [95% CI: 0.20–0.88], *p* = 0.02). After the IPTW analysis, in univariable and multivariable analyses, the use of UST continued to be significantly associated with a lower risk of relapse in both univariable (HR: 0.30 [95% CI: 0.14–0.64], *p* = 0.001) and multivariable models (HR: 0.30 [95% CI: 0.14–0.66], *p* = 0.02).

### 3.6. Adverse Events

In the UST group, arthralgia and upper respiratory infection occurred in one patient each. In the VED group, one patient discontinued VED due to arthralgia-related adverse events, and upper respiratory infections occurred in two patients (Table 5). No serious adverse events were observed.

## 4. Discussion

This study is the first to show that UST can be more effective than VED when used in maintenance with TAC therapy for the treatment of ASUC. At week 16, the clinical remission rate was significantly higher in the UST group than in the VED group, even though there were tendencies for more bloody stools at the time of starting TAC and lower albumin levels at the time of administration in the UST group. This result highlights the potential superiority of UST in achieving sustained remission during the early stages of maintenance therapy.

Several reports have demonstrated the effectiveness of UST and VED maintenance therapy after induction with a CNI [13,14,15]. However, these reports were not exclusively limited to patients with ASUC, and none of them conducted a direct comparison between the UST and VED groups in terms of efficacy. In one study, comprising a large proportion of patients with ASUC (79%), the clinical remission rate was 38% among all patients at week 14 of VED administration after CNI induction therapy [13]. Another study, comprising 76% of patients with ASUC, reported a clinical remission rate of 50% among all patients [14]. Our study is the first to focus exclusively on patients with ASUC, and the clinical remission rate was 40.9% and 51.8% after the IPTW analysis at week 16, even though all of the patients in our study had ASUC. Thus, the clinical remission rate in the VED group in our study is consistent with that reported in previous studies. Regarding UST, previous reports on its efficacy in maintenance with a CNI are limited. One study reported that 9 out of 10 patients with ASUC achieved a clinical response or remission after 6 months UST treatment [15].

The UNIFI (Ustekinumab Induction and Maintenance Therapy for Ulcerative Colitis) trial showed that 60% of patients who did not respond to UST at week 8 achieved a delayed clinical response at week 16 [12]. In contrast, in the GEMINI 1 study [11], post hoc analysis of patients with no response to VED at week 6 yielded a clinical remission rate at week 14 of 15.0%, which is comparable to the rate in the placebo group [23]. In the present study, the administration of UST after induction with TAC was significantly superior to the administration of VED in terms of achieving remission at 16 weeks. After the IPTW analysis, the remission rate at 8 weeks was significantly higher in the UST group than in the VED group. We hypothesize that the difference in remission rates may reflect the inherent differences in the time-dependent efficacy profiles of UST and VED, as highlighted by the UNIFI and GEMINI trials [9,10] as well as the synergistic effect of the TAC and UST maintenance.

A CNI combined with UST may offer an effective approach to achieving immunosuppression from an immunopathologic perspective. CNIs target the immune system by decreasing activated regulatory T (Treg) cells and effector T cells [24], and UST inhibits the activation of Th17 cells [25]. The interplay between Treg cells and Th17 is antagonistic, which means that altering the balance between these two cell types is vital for regulating autoimmunity [26]. The suppression of Th17 responses by UST, together with the inhibition of Treg and effector T cells activation by TAC, might work as a well-balanced immunosuppressive response. This balanced modulation of the immune system may explain the enhanced efficacy of maintenance therapy in patients treated with UST and TAC in the long term.

In the two previous studies of patients who received VED as maintenance therapy after CNI induction, 43% and 44% of patients were still on VED at 1 year, respectively [11,12]. We reported similar findings. Although previous research suggested a high persistence rate for UST following CNI induction [15], the persistence rates were not directly compared between UST and VED in prior studies, making ours the first to make this comparison. In addition, the use of UST significantly had a low hazard of relapse in the univariable and multivariable analyses. This reinforces the idea that the higher persistence rate of UST after TAC induction, as compared with VED, as observed in our study, indicates that UST may be a better maintenance therapy than VED. Despite the significantly higher persistence rate for UST and significantly lower hospitalization rate compared with VED after the IPTW analysis, the reasons for the comparable surgical-free remain unclear. One possible explanation is that patients in the VED group, of whom few had previously received biologics or JAK inhibitors, switched to these treatments sooner, thereby avoiding hospitalization and surgery. This possibility is supported by the diverse biologics used as subsequent therapy in the VED group (Figure 7). In the present study, one patient discontinued VED due to arthralgia, which disappeared after discontinuing VED. No patients treated with UST discontinued treatment due to adverse events. Furthermore, no serious adverse events occurred in either the UST or VED groups. These findings indicate that both treatments were generally well tolerated.

Our study has several limitations. First, the single-center nature of this study potentially limits the generalizability of our findings to different patient populations and clinical practices. Second, although we effectively utilized clinical remission as the primary outcome, we did not perform standardized endoscopic reassessment after treatment. As mucosal healing is a key therapeutic goal in the management of ASUC, the absence of follow-up endoscopic data limits our ability to evaluate the depth and durability of response. In addition, fecal calprotectin was not measured in all patients during follow-up in our study. Third, the absence of detailed criteria for selecting UST or VED as maintenance therapy after TAC-induced improvement, which were instead chosen at the doctor’s discretion, raises the possibility of selection bias. Fourth, because all data were retrospectively collected from the patients’ clinical medical records, we could not fully adjust for unmeasured or unknown confounding factors. Fifth, the sample size was too small to perform full statistical adjustment for background differences, which may increase the risk of type I error. Even after the IPTW analysis, residual imbalances persisted, particularly in sex and disease extent (pancolitis). Important future studies with larger cohorts are warranted. Sixth, only two colectomies were reported (one in each group), which is lower than typical rates reported for ASUC. This may be partly explained by the exclusion of patients who underwent emergency colectomy due to the failure of TAC induction therapy (as shown in Figure 1). Another possible explanation is that, in cases where UST or VED failed, escalation to advanced therapies (e.g., JAK inhibitors or switching to a different biologic) was effective and further delayed the need for surgery. Because only two colectomy events occurred during follow-up, our Kaplan–Meier analyses are underpowered and should be interpreted with caution. Seventh, because UST was introduced at our center more recently than VED due to a two-year difference in insurance coverage timing, patients in the UST cohort had a shorter observation window, leading to earlier censoring in Kaplan–Meier analyses. This differential follow-up may slightly underestimate long-term event rates in the UST group. Finally, although infectious triggers have been reported in approximately 15–30% of inflammatory bowel disease flares [27,28], our infection screening did not incorporate CMV PCR (plasma or tissue). As serology alone cannot reliably exclude the possibility of concomitant active CMV colitis, this limitation may have introduced uncertainty in the assessment of disease severity.

Addressing these limitations in future research, possibly through multicenter, randomized controlled trials and longer follow-up periods, as well as exploring appropriate biomarkers [29], would strengthen the evidence base for the comparative effectiveness of these therapies for managing ASUC.

## 5. Conclusions

The present study demonstrated that patients in the UST group had a significantly higher remission rate at week 16 than those in the VED group. In addition, the biologic persistence rate was higher in the UST group. Therefore, UST appears to be a superior option to VED when used as maintenance therapy after TAC-induced improvement for managing ASUC, particularly in terms of remission and persistence rates.

## Figures and Tables

**Table 1:** Baseline clinical characteristics at admission before TAC induction in patients later treated with UST or VED.

	UST (n = 29)	VED (n = 22)	*p* Value
Partial Mayo score, median [IQR]	9.0 [9.0–9.0]	9.0 [9.0–9.0]	1.00
Number of bloody stools, median [IQR]	10.0 [8.3–12.0]	8.00 [6.00–10.00]	0.01
Heart rate [/min], median [IQR]	99.5 [88.8–102.0]	96.0 [87.0–106.0]	0.65
Body temperature [°C], median [IQR]	37.1 [36.6–37.7]	37.1 [36.9–37.8]	0.31
CRP [mg/L], median [IQR]	48.8 [24.9–74.7]	44.5 [24.4–64.6]	0.89
WBC [/μL], median [IQR]	9660.0 [7680.0–11,095.0]	11,220 [6975–12,935]	0.52
Hb [g/dL], median [IQR]	11.0 [9.6–12.1]	11.8 [11.1–13.0]	0.26
Plt [×10^4^/μL], median [IQR]	44.0 [32.7–48.7]	40.9 [33.1–52.5]	0.72
Alb [g/dL], median [IQR]	3.1 [2.3–3.6]	3.2 [2.6–3.7]	0.46
Concomitant use of prednisolone [%]	7 [25.9]	5 [22.7]	1.00
Dose of prednisolone [mg], median [IQR]	70.0 [45.0–80.0]	45.00 [22.5–60.0]	0.13

Abbreviations: JAK, Janus kinase; CRP, C-reactive protein; WBC, white blood cell count; Hb, hemoglobin; Plt, platelet count; Alb, albumin.

**Table 2:** Demographic and clinical characteristics of patients treated with UST or VED maintenance therapy after TAC induction therapy IPTW-unweighted analysis.

IPTW-Unweighted	UST (n = 29)	VED (n = 22)	Overall (n = 51)	*p* Value	SMD
Age, years, median [IQR]	35.0 [27.0, 54.0]	35.0 [29.3, 50.8]	35.0 [28.0, 53.0]	0.83	0.10
Males, n [%]	19 [65.5]	8 [36.4]	27 [52.9]	0.07	0.61
Disease type, [pancolitis] [%]	29 [100.0]	19 [86.4]	48 [94.1]	0.15	0.56
Concomitant use of immunomodulatory drug [%]	11 [37.9]	10 [45.5]	21 [41.2]	0.80	0.15
Prior medication of biologics or JAK inhibitor [%]	21 [72.4]	12 [54.5]	33 [64.7]	0.30	0.38
Steroid refractory [%]	28 [96.6]	18 [81.8]	46 [90.2]	0.23	0.49
Duration of TAC therapy, median [IQR]	185.0 [96.0, 215.0]	149.5 [147.8, 242.8]	168.0 [100.0, 239.5]	0.14	0.23
The time from the initiation of TAC to the start of maintenance therapy, median [IQR]	35.0 [21.0, 45.0]	46.5 [29.3, 72.8]	36.0 [21.0, 65.0]	0.31	0.43
Partial Mayo score, median [IQR]	3.0 [2.0, 6.0]	3.0 [1.0, 5.0]	3.0 [1.0, 5.0]	0.50	0.07
CRP [mg/L], median [IQR]	1.0 [0.4, 2.0]	2.0 [0.5, 3.0]	1.0 [0.4, 2.0]	0.76	0.32
WBC [/μL], median [IQR]	6020.0 [4330.0, 7030.0]	6670.0 [5475.0, 7852.5]	6400.0 [4920.0, 7305.0]	0.20	0.39
Hb [g/dL], median [IQR]	10.2 [9.4, 11.9]	11.0 [9.8, 12.3]	10.3 [9.5, 12.1]	0.34	0.22
Plt [×10^4^/μL], median [IQR]	29.7 [26.7, 36.7]	32.8 [29.2, 38.8]	32.1 [26.8, 37.4]	0.14	0.53
Alb [g/dL], median [IQR]	3.5 [3.0, 4.1]	4.1 [3.6, 4.2]	3.8 [3.2, 4.2]	0.06	0.60

Abbreviations: IPTW, inverse probability of treatment weighting; SMD, standardized mean difference.

**Table 3:** Demographic and clinical characteristics of patients treated with UST or VED maintenance therapy after TAC induction therapy IPTW-weighted analysis.

IPTW-Weighted	UST (n = 27.4)	VED (n = 22.0)	Overall (n = 49.4)	*p* Value	SMD	Std-Diff
Age, years, median [IQR]	37.8 [27.3, 56.9]	32.7 [29.5, 50.2]	36.8 [28.2, 55.5]	0.84	0.03	
Males, n [%]	18.8 [68.8]	8.1 [36.8]	26.9 [54.5]	0.04	0.68	0.28
Disease type, [pancolitis] [%]	27.4 [100.0]	20.7 [94.1]	48.1 [97.4]	0.08	0.35	
Concomitant use of immunomodulatory drug [%]	10.3 [37.6]	8.9 [40.5]	19.2 [49.5]	0.82	0.07	
Prior medication of biologics or JAK inhibitor [%]	16.8 [61.3]	14.2 [64.6]	31.0 [62.8]	0.84	0.06	0.05
Steroid refractory [%]	24.3 [88.7]	19.8 [90.0]	44.1 [89.3]	0.91	0.04	
Duration of TAC therapy, median [IQR]	139.0 [91.2, 208.7]	175.5 [149.0, 244.3]	161.6 [97.2, 237.3]	0.15	0.28	
The time from the initiation of TAC to the start of maintenance therapy, median [IQR]	24.5 [20.6, 45.0]	40.1 [18.1, 59.3]	31.6 [20.1, 53.4]	0.54	0.24	
Partial Mayo score, median [IQR]	3.0 [1.2, 4.4]	3.0 [0.4, 5.0]	3.0 [1.0, 5.0]	0.73	0.15	0.12
CRP [mg/L], median [IQR]	1.1 [0.4, 2.0]	2.0 [0.5, 4.0]	1.2 [0.4, 4.0]	0.65	0.50	0.13
WBC [/μL], median [IQR]	6460.0 [4317.8, 7289.7]	6370.9 [5302.8, 7790.0]	6395.3 [4929.8, 7723.8]	0.44	0.28	
Hb [g/dL], median [IQR]	10.8 [9.4, 12.0]	10.8 [8.8, 12.3]	10.9 [9.4, 12.3]	0.90	0.01	
Plt [×10^4^/μL], median [IQR]	29.8 [26.7, 35.5]	32.2 [29.0, 38.8]	30.9 [27.1, 37.0]	0.12	0.52	
Alb [g/dL], median [IQR]	3.4 [3.0, 4.0]	3.9 [3.5, 4.1]	3.7 [3.2, 4.1]	0.10	0.51	0.01

Abbreviations: Std-diff, standardized difference.

**Table 4:** Risk factors for relapse.

**IPTW-Unweighted Cox Proportional Hazards Model**	**Univariable**			**Multivariable**		
**Characteristics**	**HR**	**95% CI**	***p*** **Value**	**HR**	**95% CI**	***p*** **Value**
The use of UST as maintenance therapy	0.39	0.19–0.81	0.001	0.42	0.20–0.88	0.02
The days between the start of TAC and the administration of the biologics	1.01	1.00–1.01	0.05	1.00	1.00–1.01	0.13
**IPTW-Weighted Cox Proportional Hazards Model**	**Univariable**			**Multivariable**		
**Characteristics**	**HR**	**95% CI**	***p*** **Value**	**HR**	**95% CI**	***p*** **Value**
The use of UST as maintenance therapy	0.30	0.14–0.64	0.001	0.30	0.14–0.66	0.02
The days between the start of TAC and the administration of the biologics	1.00	1.00–1.01	0.42	1.00	1.00–1.01	0.34

Abbreviations: HR, hazard ratio.

**Table 5:** Adverse events between UST and VED.

	UST (n = 29)	VED (n = 22)
Arthralgia [%]	1 (3.4)	1 (4.5)
Upper respiratory tract infection	1 (3.4)	2 (9.0)

## Data Availability

The data that support the findings of this study are not publicly available due to privacy concerns but are available from the corresponding author upon reasonable request.

## Supplementary Materials

The following supporting information can be downloaded at: https://www.mdpi.com/article/10.3390/jcm14155588/s1, Supplementary Table S1: Summary of International Guidelines on Rescue Therapy for Steroid-Refractory ASUC.

## Abbreviations

ASUC, acute severe ulcerative colitis; TAC, tacrolimus; UST, ustekinumab; VED, vedolizumab; CNI, calcineurin inhibitor; JAK, Janus kinase; CRP, C-reactive protein; HR, hazard ratio; CI, confidence interval; IPTW, inverse probability of treatment weighting.
