# Supplementary material for: Comparative Effectiveness of Ustekinumab and Vedolizumab as Maintenance Therapy After Tacrolimus-Induced Improvement in Patients with Acute Severe Ulcerative Colitis: A Retrospective Cohort Study
Source: J Clin Med. 2025 Aug 7;14(15):5588. doi: 10.3390/jcm14155588 (PMC12347027; doi:10.3390/jcm14155588)
Supplement: Supplementary file 1 [file jcm-14-05588-s001.zip › jcm-3709968-supplementary.pdf]

Supplementary Table S1. Summary of International Guidelines on Rescue Therapy for Steroid-Refractory ASUC.

Guideline	Extracted / Paraphrased Statement (Steroid-Refractory ASUC)
JSGE 2021 [3]	"Oral tacrolimus is recommended in patients with severe UC who do not respond to intravenous corticosteroids."
ECCO [4]	"Either infliximab or cyclosporine should be used in adult patients with steroid-refractory ASUC. " "Third-line sequential rescue therapies with calcineurin inhibitors [cyclosporine or tacrolimus] in ASUC refractory to corticosteroid therapy may delay the need for colectomy"
BSG [5]	"Patients with ASUC not responding to at least 3 days of IV corticosteroids, as judged by a suitable scoring system, should be treated with rescue therapy in the form of intravenous infliximab or ciclosporin."
AGA	"In patients with ASUC failing to adequately respond to intravenous corticosteroids by 3–5 days we recommend medical rescue therapy with infliximab or cyclosporine"

Abbreviations: ASUC, acute severe ulcerative colitis; JSGE, Japanese Society of Gastroenterology; ECCO, European Crohn's and Colitis Organisation;BSG, British Society of Gastroenterology; AGA, American Gastroenterological Association.
